# Cerebritis: An unusual complication of *Klebsiella pneumoniae*

**DOI:** 10.4103/0972-5229.53116

**Published:** 2009

**Authors:** Mainak Majumdar, David C. Simes1, Ramesh D. Prabha1

**Affiliations:** **From:** Department of Critical Care, Latrobe Regional Hospital, Traralgon, Vic 3844, Intensive Care Unit, Fremantle Hospital, Alma Street, Fremantle, WA6160, Australia

**Keywords:** Alcohol, cerebritis, *Klebsiella*

## Abstract

Cerebritis is part of a continuum of brain infection and is difficult to diagnose. Cerebritis caused by *Klebsiella* in immunocompetent adults without predisposing factors such as neurosurgery or penetrating brain injury has not been reported before. We report a case of *Klebsiella* cerebritis in an adult patient with a proven extracranial focus of infection. We suggest considering cerebritis as a differential diagnosis for altered level of consciousness in patients of severe sepsis, even if an extracranial source of infection is proven.

## Introduction

Cerebritis without a history of penetrating head trauma or neurosurgery is a rarely suspected cause of coma. Bacteria gain entry to brain tissue and cause infection either by direct spread or through hematogenous seeding.[1] In Gram-negative CNS infections, a primary focus of infection may be found in neonates and in trauma and neurosurgical patients, but in adults without antecedent surgery, there will be no primary focus of infection detected in up to 60% of cases.[2] Further, sedation in the intensive care unit (ICU) may mask progressive brain insult. Thus, cerebritis may be well advanced before measures are taken to curb its progression.

## Case Report

A 48-year-old lady presented with increasing confusion following a three-day history of fever, shortness of breath, and productive cough. Her background included heavy smoking, heavy ethanol use, and two prior hospital admissions for pneumonia and exacerbation of chronic obstructive airways disease.

At presentation she was febrile, delirious, and tachypneic with low oxygen saturations, relative hypotension, and new onset atrial fibrillation with a rapid ventricular response. Her chest X-ray revealed dense right upper and middle lobe consolidation. Initial treatment consisted of oxygen therapy, intravenous fluid resuscitation, noninvasive ventilation, and antibiotics (ceftriaxone and azithromycin) for her community acquired pneumonia. She reverted to sinus tachycardia after treatment with digoxin. She was prescribed benzodiazepines for apparent alcohol withdrawal agitation.

She was transferred to our institution after two days in established multiorgan failure with shock, respiratory failure, coagulopathy. Invasive organ support was initiated with intubation, mechanical ventilation, vigorous fluid resuscitation, vasopressors, and inotropes. Blood and sputum cultures taken at the time of her initial presentation grew *Klebsiella pneumoniae* sensitive to all antibiotics except amoxicillin. Antibiotics were changed to ceftriaxone and ciprofloxacin. Human immunodeficiency virus (HIV) screen was negative. Echocardiography revealed normal valve function, with no sign of vegetations and normal chamber size and function.

By day 3 in the ICU, her gas exchange had improved. Though tachycardic and febrile, she maintained sinus rhythm and sustained a mean arterial pressure above 70 mm Hg without vasopressor support. Sedation was then discontinued for the first time since intubation. Her pupils were noted to react to light but she was unresponsive to painful stimuli. There was no further change to her neurological status 24 hours after stopping sedation.

**Figure 1:**
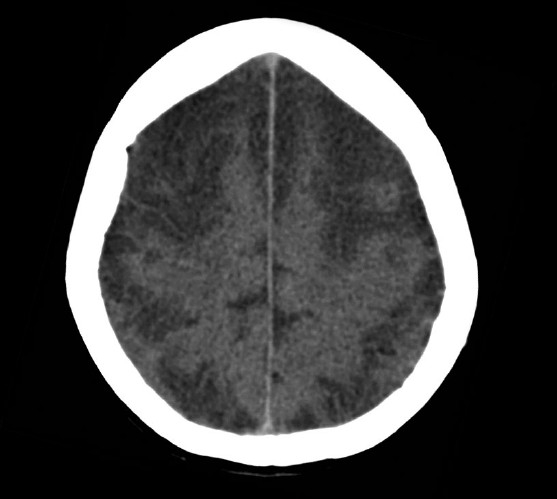
CT brain with contrast, day 5 in ICU

**Figure 2:**
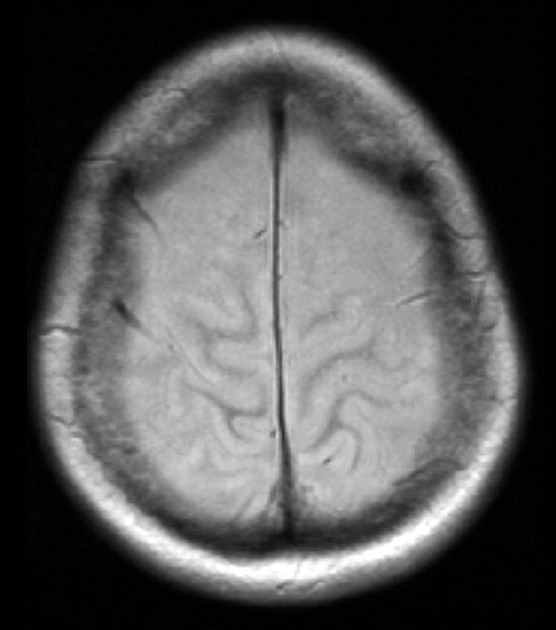
MRI brain, day 5 in ICU

**Figure 3:**
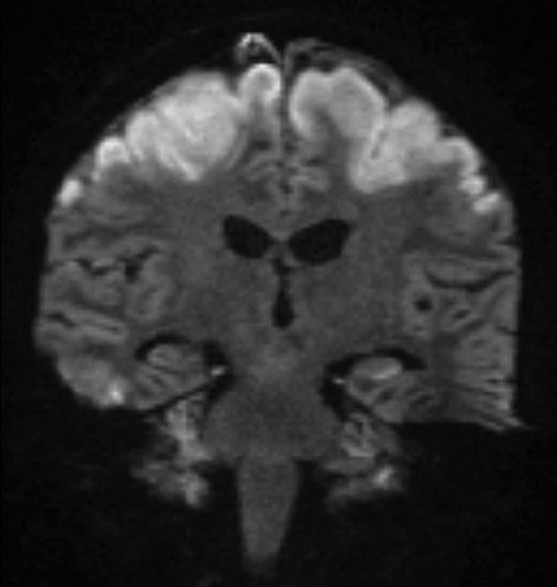
Diffusion weighted MRI, day 5 in ICU

On day 5, she had a focal seizure limited to her left arm, lasting less than a minute. Her pupils were equal but noted to be unreactive, her gaze was divergent and a flaccid quadriplegia and absence of deep tendon reflexes were noted for the first time. An urgent CT brain demonstrated advanced cerebral injury [Figure 1], initially reported as extensive cortical necrosis of the frontal, parietal, and temporal lobes. To further define the extent of the inflammatory process, magnetic resonance imaging (MRI) [Figures 2 and 3] was performed. There was widespread nonvascular territory cortical infarction, particularly over both convexities. Given the additional patchy involvement of cortex elsewhere, particularly within the posterior fossa, hippocampi, and occipital horns of the lateral ventricles, the overall pathology was deemed to be cerebritis with hyperaemia and associated meningitis. There were no features suggestive of focal cerebral abscess and the dural venous sinuses appeared patent.

Neurosurgical advice was sought. It was felt that bifrontal craniotomy to reduce intracranial pressure would confer little benefit. In view of ongoing coagulopathy and thrombocytopenia, the risks of placing an intraventricular drain were considered to outweigh any benefits in guiding therapy. Spinal fluid was not sought for the same reasons.

**Figure 4:**
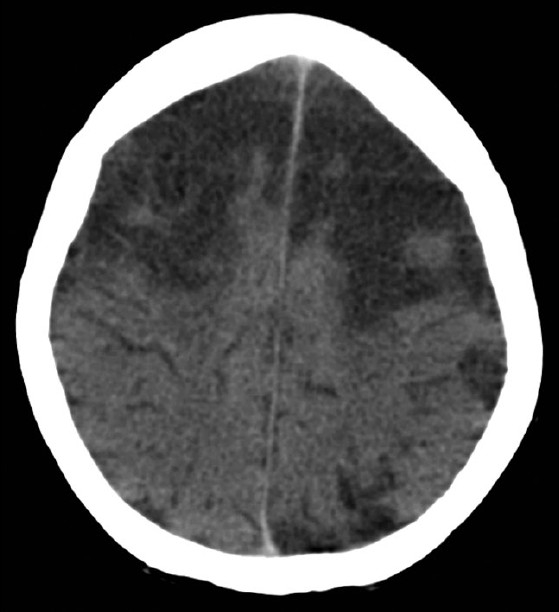
CT brain with contrast, day 6 in ICU, deteriorating neurologic status

On day 6 the patient developed dilated and fixed pupils, absent corneal reflexes, and absent plantar reflexes bilaterally. She still had a cough reflex and took spontaneous breaths on pressure support. A second CT brain [Figure 4] showed increasing areas of low attenuation within the frontoparietal and superior occipital cerebral cortex with evidence of mass effect.

A meeting was held with her family to inform them of her extremely poor prognosis. In keeping with her family's understanding of her previously expressed wishes, mechanical ventilation was withdrawn and palliative measures instituted. The patient died two days later.

## Discussion

Our patient was a 48-year-old female with good premorbid level of function, few hospital admissions, and no obvious risks for compromised or suppressed immune function. We did not expect her to develop a fatal CNS infection, while the extracranial focus of infection was demonstrably resolving (radiologically and in terms of measured gas exchange) on two antibiotics to which the pathogen was susceptible *in vitro*.

However, a review of literature suggests *Klebsiella* may possess unique infective and proinflammatory properties[3] that render it particularly virulent in the alcoholic host.[4] There is emerging laboratory data on murine models demonstrating reduced cytokine release (IL-17) in animals pretreated with alcohol prior to exposure to *Klebsiella*. The alcohol-treated animals also demonstrated distinctly worse clinical appearances.[5] A similar two-week alcohol exposure model showed increased mortality and decreased IL-17 production when *Klebsiella pneumoniae* was introduced to a murine pneumonia model.[6] Other studies also implicate IL-23 in the murine model.[7] Work on human endothelium and macrophage lines confirms that acute alcohol intoxication significantly inhibits lymphocyte expression during pulmonary inflammation.[8]

*Klebsiella* has been reported as a CNS pathogen with increasing frequency, with no improvement in prognosis over a 15-year period despite the availability of newer antibiotics. Particularly high mortality with *Klebsiella* septicemia and CNS spread has been noted.[9]

Diagnosing cerebritis requires a combination of high index of suspicion, diligent neurological examination during regular sedation holidays, and appropriate imaging studies. MRI images reflect the duration and severity of the illness.[10]

Given our experience, cerebritis should be considered a differential diagnosis in patients with a history of heavy ethanol consumption in the setting of *Klebsiella* sepsis and altered level of consciousness. Unfortunately, early diagnosis may only serve to allow appropriate counselling to relatives, without conferring a mortality advantage.
